# Does Apolipoprotein E polymorphism play a role in familial Alzheimer’s Dementia

**DOI:** 10.1002/alz.087257

**Published:** 2025-01-03

**Authors:** Pratibha Vinod, Somdatta Sen, Pavithra Jayasankar, Meghana Janardhanan, Pradip Paul, Biju Viswanath, Sanjeev Jain, Vijaykumar Harbishettar, Palanimuthu Thangaraju Sivakumar, Meera Purushottam

**Affiliations:** ^1^ National Institute of Mental Health and Neurosciences, Bangalore, Karnataka India; ^2^ Department of Molecular Genetics(NIMHANS), Bangalore, Karnataka India; ^3^ Department of Molecular Genetics (NIMHANS), Bangalore, Karnataka India; ^4^ National Institute of Mental Health and Neurosciences (NIMHANS), Bangalore, Karnataka India; ^5^ Department of Psychiatry, National Institute of Mental Health and Neurosciences (NIMHANS), Bengaluru, Karnataka India; ^6^ NIMHANS, Bangalore India; ^7^ National Institute of Mental Health and Neurosciences (NIMHANS), Bengaluru, India), Bengaluru India; ^8^ National Institute of Mental Health and Neurosciences, Bangalore India; ^9^ National Institute of Mental Health and Neurosciences (NIMHANS), Bangalore India; ^10^ Molecular Genetics Lab Department of Psychiatry, National Institute of Mental Health and Neurosciences(NIMHANS), India, Bengaluru, Karnataka India

## Abstract

**Background:**

*Apo E4* is the best studied genetic risk factor for sporadic Alzheimer’s disease. *Apo E2* homozygosity is associated with a lower risk of Alzheimer’s disease. While rare and common variants in genes encoding APP metabolism are strongly linked to familial dementia, however family history and *ApoE 4* genetic risks have been found to co‐occur.

Aim to evaluate the frequency of *Apo E* polymorphisms and their relationship with the of family history and clinical characteristics in a cohort of 483 patients with Alzheimer’s dementia.

**Method:**

Persons with Alzheimer’s dementia were identified from a tertiary care center in India. Their family history status, and detailed clinical characteristics were collected. Genotyping was done at the apolipoprotein E (*Apo E*) locus using real time PCR.

**Result:**

Of the 483 patients analyzed; 142 patients had a family history of Alzheimer’s dementia in at least one first degree relative. Absence of *Apo E2* homozygosity was noted in the sample. Patients with family history had a higher likelihood of an *Apo E4* carrier status (OR = 1.6071; Confidence Interval 1.0840 to 2.3829 p = 0.00182).

The *Apo E4* frequency in the overall sample was 25.05%.

**Conclusion:**

There is a correlation between *Apo E4* carrier status and the likelihood of a positive family history. It remains to be known whether family history effects are separable from *Apo E4* related effects.

